# Potential Therapeutic Actions of Flavonoids Present in Propolis That Modulate Vascular Endothelial Growth Factor Signaling to Regulate Angiogenesis

**DOI:** 10.1002/ptr.70029

**Published:** 2025-08-09

**Authors:** Yipeng Lin, Janney Z. Wang, Yihe Niu, Ling Zhu, Rujee K. Duke, Colin C. Duke, Michael Murray, Fanfan Zhou

**Affiliations:** ^1^ Molecular Drug Development Group, Sydney Pharmacy School, Faculty of Medicine and Health The University of Sydney Sydney New South Wales Australia; ^2^ Macular Research Group, Save Sight Institute, Faculty of Medicine and Health The University of Sydney Sydney New South Wales Australia; ^3^ School of Medical Sciences, Faculty of Medicine and Health The University of Sydney Sydney New South Wales Australia

**Keywords:** angiogenesis, flavonoids, inflammation, intraocular neovascular syndromes, propolis, solid tumour, VEGF

## Abstract

Propolis is a plant‐derived substance collected by honeybees that has a range of potential therapeutic applications. Propolis consists of resins, waxes, and fatty acids, as well as essential oils and other organic compounds. The pharmacologically active components of propolis include phenols and flavonoids, among others. Flavonoids that are found in propolis include chrysin, quercetin, galangin, kaempferol, and apigenin. Angiogenesis is the growth of new blood vessels from endothelial cell precursors. Angiogenesis is important in normal physiology and development. Pathological angiogenesis is widely implicated in human diseases, such as retinal diseases, cancers, and inflammatory diseases; currently, many of these conditions lack effective treatments. The process of angiogenesis is modulated by pro‐angiogenic factors, in particular, Vascular Endothelial Growth Factor (VEGF). Flavonoids, including several that are present in propolis, have been found to inhibit angiogenesis by attenuating VEGF signaling. Although promising, such findings are primarily based on preclinical studies, and few clinical studies have evaluated the efficacy and toxicity of flavonoids in vivo. This review outlines the therapeutic potential of essential flavonoids from propolis that may have value as anti‐angiogenic agents by modulating VEGF signaling. Overall, the findings suggest that flavonoids that are present in propolis are potential agents for the treatment of human diseases in which pathological angiogenesis is activated.

**Abbreviations**
AMD: age‐related macular degeneration
CAPE: caffeic acid phenethyl ester
CNV: choroidal neovascularization
CVDs: cardiovascular diseases
DAG: diacylglycerol
DR: diabetic retinopathy
eNOS: endothelial nitric oxide synthase
ER: estrogen receptor
ERK: extracellular signal‐regulated kinase
HCC: hepatocellular carcinoma
HIF‐1α: hypoxia inducible factor‐1 α
HUVEC: human umbilical vein endothelial cell
IL‐1β: interleukin 1 β
IL‐6: interleukin‐6
LPS: lipopolysaccharide
MCF‐7: Michigan cancer foundation‐7
MDA‐MB‐231: MD Anderson‐metastatic breast‐231
MEK: mitogen‐activated protein kinase
mTOR: mammalian target of rapamycin
NF‐κB: nuclear factor‐κ B
NO: nitric oxide
P70S6K: p70S6 kinase
PI3K: phosphoinositide 3‐kinase
PIGF: placental growth factor
PIN: prostatic intraepithelial neoplasia
PKB: protein kinase B
PKC: protein kinase C
PLCγ: phospholipase C γ
RA: rheumatoid arthritis
ROS: reactive oxygen species
STAT3: signal transducer and activator of transcription 3
TNF‐α: tumor necrosis factor α
VEGF: vascular endothelial growth factor

## 1 Introduction

New blood vessel formation is a two‐step mechanism involving angiogenesis and vasculogenesis (Poole and Coffin 1989; Laschke et al. 2011). Vasculogenesis is the emergence of a vascular network following the differentiation of endothelial progenitor cells, whereas angiogenesis is the development of new capillaries from existing blood vessels (Zygmunt et al. 2003; Hoff and Machado 2012). These processes are important in the adult vasculature as well as in the embryonic and fetal periods (Kässmeyer et al. 2009). Solid cords are formed by the rapid organization and coalescence of angioblasts at different embryonic locations, forming an initial plexus of blood vessels which then transforms into tubes to carry blood (Xu and Cleaver 2011). The relocation of endothelial cells occurs after degradation of the basement membrane, followed by migration to produce new capillaries (Sedighi et al. 2023).

Angiogenesis is a central component of many physiological processes, encompassing not only embryonic development but also tissue regeneration following surgical procedures or traumatic injuries (Yoo and Kwon 2013). In angiogenesis, the circulatory system is remodeled, which enables the maintenane of blood pressure, immune responses, and tissue oxygenation (Patel‐Hett and D'Amore 2011). Angiogenesis is also important in pathological processes of disease progression, such as in cancers, rheumatoid arthritis, proliferative retinopathy, age‐related macular degeneration (AMD), hemangiomas, malformations, and psoriasis (Ferrara 2002; Zhao et al. 2010). Thus, inflammatory cells migrate to the site of injury and trigger the local production of pro‐angiogenic factors by endothelial cells, tumor cells, inflammatory cells, platelets, and smooth muscle cells (Melincovici et al. 2018).

Intraocular neovascularization is a major cause of vision impairment (Campochiaro 2013). Pathological neovascularization can trigger leakage and hemorrhage, followed by fibrous proliferation that leads to vision impairment (Zhou et al. 2017). In cancers, angiogenesis increases drug, nutrient, growth hormone, and oxygen delivery that contribute to tumour progression and metastasis (Al‐Ostoot et al. 2021). It has been demonstrated that both the initiation and regulation of angiogenesis are responsive to changes in the local balance of pro‐angiogenic factors and angiogenesis inhibitors (Zygmunt et al. 2003). Positive regulators of angiogenesis can be broadly categorized into three classes (Klagsbrun and Moses 1999). One includes the vascular endothelial growth factor (VEGF) family and angiopoietins, which specifically target endothelial cells (Liekens et al. 2001). Another class comprises direct‐acting molecules such as fibroblast growth factor‐2 (Bussolino et al. 1996). The third class includes indirect‐acting factors that promote angiogenesis by modulating the release of direct‐acting factors from endothelial cells, macrophages, or tumour cells (Liekens et al. 2001); these factors include tumour necrosis factor alpha (TNF‐α) (Jackson et al. 1997). VEGF‐dependent and VEGF‐independent mechanisms have been explored for their application in angiogenesis‐related diseases (Duval et al. 2003). For instance, the target‐selective transactivation of estrogen receptors (ER) may result in bimodal regulation of angiogenesis, leading to cardiovascular protective or cancer preventive effects (Liu et al. 2016; Iorga et al. 2017). In breast cancer, Notch signaling and its crosstalk with other pathways contribute to tumour growth, metastasis, and angiogenesis. Thus, Notch antagonists have significant clinical potential (Zhou et al. 2013). Moreover, endogenous inhibitors of angiogenesis have also been identified, such as thrombospondin‐1 (Farnoodian et al. 2018) and pigment epithelium‐derived factor (Dawson et al. 1999). VEGF is perhaps the most important and well‐studied regulator of angiogenesis and therefore is the principal focus of this review (Zygmunt et al. 2003; Ferrara and Davis‐Smyth 1997; Karamysheva 2008).

## 2 VEGF Signaling in Angiogenesis

VEGF expression is activated in hypoxia (Nourizad et al. 2023; Elebiyo et al. 2022). The VEGF family includes VEGF‐A, VEGF‐B, VEGF‐C, VEGF‐D, and placental growth factor (PIGF) (Nourizad et al. 2023; Elebiyo et al. 2022; Olsson et al. 2006; Ferrara and Ten Adamis 2016). These are secreted dimeric glycoproteins with molecular weights of ~40 kDa (Olsson et al. 2006). VEGF isoforms are ligands for specific VEGF receptor family members that activate physiological and pathological angiogenesis (Table 1). Multiple signaling networks are triggered by VEGF‐dependent activation of VEGF receptor signaling cascades, which promote the survival, mitogenesis, migration, and differentiation of endothelial cells (Hicklin and Ellis 2005). Among all VEGF family members, VEGF‐A plays a key role in angiogenesis and vasculogenesis (Zhao et al. 2010; Shin et al. 2016; Geng et al. 2013). The interactions between VEGF‐A and its receptors VEGFR1 and VEGFR2 are important in physiological and pathological angiogenesis (Zhao et al. 2010; Abhinand et al. 2016; Shibuya 2011).

**TABLE 1:** VEGF receptors and their VEGF ligands.

VEGF receptor	VEGF ligands	Physiological and/or pathological functions of VEGF receptors	References
VEGFR1	VEGF‐A, VEGF‐B, PIGF	Regulation of cell migration and proliferation	(Kerber et al. 2008; Sakurai et al. 2005)
VEGFR2	VEGF‐A, VEGF‐C, VEGF‐D	Regulation of angiogenesis and vascular permeability	(Zhao et al. 2010; Abhinand et al. 2016; Shibuya 2011)
VEGFR3	VEGF‐C, VEGF‐D	Regulation of angiogenesis and lymphangiogenesis	(Cao et al. 1998; Deng et al. 2015; Benedito et al. 2012)

**FIGURE 1:**
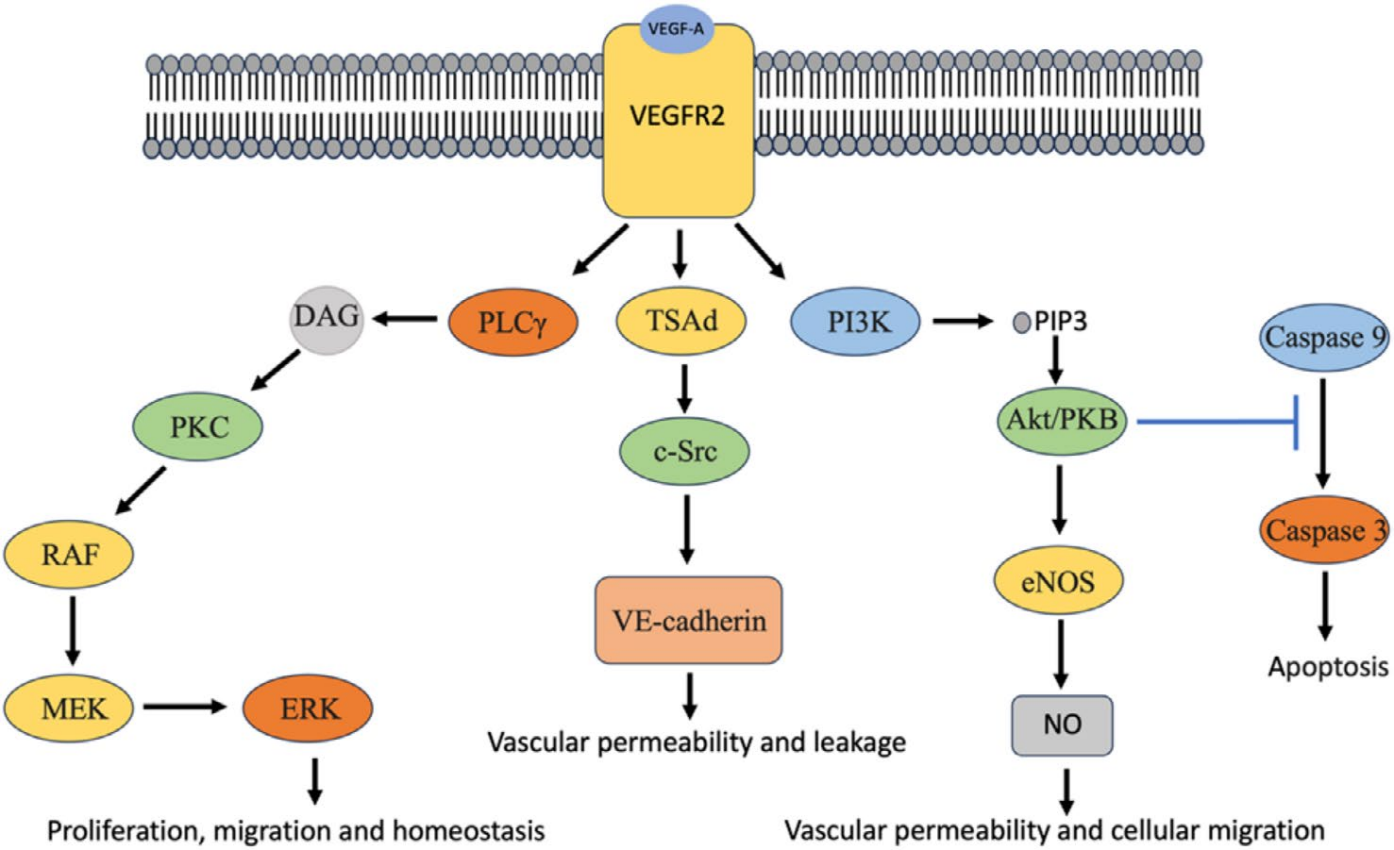
Overview of VEGF signaling in cells. The binding of VEGF‐A to VEGFR2 activates PLCγ–PKC signaling, which activates Raf and MEK signaling and the downstream ERK pathway. These pathways modulate cell proliferation, migration, and homeostasis. The binding of VEGF‐A to VEGFR2 can also induce the activation of PI3K, which increases PIP3 accumulation and Akt/PKB phosphorylation, which inhibits cell apoptosis by inhibiting the phosphorylation of Caspase 9 and the activation of Caspase 3. Akt/PKB also activates eNOS, which generates NO and modulates vascular permeability and cellular migration. The interaction of VEGF‐A with VEGFR2 can also stimulate c‐SRC and VE‐cadherin to promote vascular permeability and leakage. DAG, diacylglycerol; eNOS, endothelial nitric oxide synthase; ERK, extracellular signal‐regulated kinase; MEK, mitogen‐activated protein kinase; NO, nitric oxide; PI3K, phosphoinositide 3‐kinase; PIP3, phosphatidylinositol‐3,4,5‐triphosphate; PKC, protein kinase C; PLCγ, phospholipase C γ.

Binding of VEGF‐A to VEGFR2 induces autophosphorylation of specific tyrosine residues in the VEGFR2 cytoplasmic domain (Figure 1) (Abhinand et al. 2016). The phosphorylation and activation of VEGFR2 leads in turn to the activation of PLCγ–protein kinase C (PKC) signaling (Zhang et al. 2019; Pérez‐Gutiérrez and Ferrara 2023). PLC catalyzes the hydrolysis of phosphatidylinositol 4,5‐bisphosphate, which produces diacylglycerol (DAG) that activates PKC (Lai and Feng 2004). This step activates the Raf‐activated mitogen‐activated protein kinase (MEK) and the downstream extracellular signal‐regulated kinase (ERK) pathway (Pintus et al. 2003), which regulates cell proliferation, migration, and homeostasis (Zhang et al. 2019; Pérez‐Gutiérrez and Ferrara 2023). The binding of VEGF‐A to VEGFR2 also activates PI3K, increases the accumulation of phosphatidylinositol‐3,4,5‐triphosphate (PIP3) and promotes the phosphorylation of Akt/PKB (Nilsson and Heymach 2006). Nitric oxide (NO) is generated by endothelial nitric oxide synthase (eNOS), which is also activated by the Akt/PKB pathway and increases vascular permeability and cellular migration (Cross et al. 2003).

Increased VEGF‐A expression has been observed in multiple solid tumors, including lung, breast, and gastrointestinal cancers. Thus, VEGF‐A overexpression is a potential cause of poor prognosis and increased risk of cancer recurrence (Rosen 2002). In hypoxia, including hypoxic regions of solid tumors, the dimeric transcription factor hypoxia‐inducible factor‐1 (HIF‐1) binds to a hypoxia response element in the promoter region of the VEGF gene and activates expression (Huang et al. 1996; Sutter et al. 2000). In some pathological conditions, signal transducer and activator of transcription 3 (STAT3) is also activated, which increases both the stability of HIF‐1α by inhibiting its degradation and its *de novo* synthesis, which increases HIF‐1α and VEGF gene expression (Jung et al. 2005). In addition, VEGF‐A has also been implicated in the pathogenesis of aging diseases such as senile cataractogenesis and AMD (Marneros 2016). Certain agents that modulate the VEGF cascade, including anti‐VEGF drugs, such as sorafenib (Blumenschein 2008; Takimoto and Awada 2008) and aflibercept (Stewart 2012; Chu 2009), have been approved to treat pathological angiogenesis in disease. Clinical studies have demonstrated that anti‐VEGF drugs may inhibit intraocular neovascularization (Jianjiang et al. 2014; Mintz‐Hittner and Kuffel 2008). However, most clinically used anti‐VEGF drugs are associated with an enhanced risk of local adverse reactions like intraocular inflammation, retinal detachment, increased intraocular pressure, ocular inflammation, congestion, and hemorrhage (Amadio et al. 2016). The development of better‐tolerated anti‐VEGF agents would offer clinical advantages over currently approved drugs.

## 3 Propolis and Flavonoids

Propolis (also known as bee glue) is a dark, viscous substance that honeybees collect from plants and utilize within their hives (Bankova 2005). Bees gather resin from plant buds, secretions, and other sources, and then combine it with salivary enzymes and beeswax to produce propolis (Cui et al. 2022). Propolis consists of ~45% resins, ~30% waxes and fatty acids, ~10% essential oils, ~5% pollen, and ~10% organic compounds and minerals (Ghisalberti et al. 1978). The principal chemical components of propolis are phenolics, flavonoids, terpenes, alcohols, sugars, esters, and others (Cui et al. 2022; Patel 2016). The composition of propolis varies considerably due to geographical location, local plant varieties, and types of honeybees. Thus, the phytochemical and biological profile of propolis is dependent on ethnobotanical sources (de Almeida‐Junior et al. 2023). For example, poplar is the main botanical source of propolis in Egypt, and the clusia plant is a major source in Cuba (Ristivojević et al. 2015; Wagh 2013). Because bees can travel between geographical regions, the composition of propolis may be derived from multiple botanical sources. The diverse composition of propolis also leads to different potential clinical applications. Some propolis extracts contain over 100 flavonoids, such as luteolin, galangin, chrysin, kaempferol, myricetin, quercetin, apigenin, and others (Table 2) (Huang et al. 2014). As key constituents, flavonoids play pivotal roles in the pharmacological activities of propolis (Huang et al. 2014; Chan et al. 2013), which include antioxidative (Gregoris and Stevanato 2010), antifungal (Freires et al. 2016), and anti‐inflammatory properties (Bueno‐Silva et al. 2013). Other chemical constituents of propolis also exhibit a wide range of biological activities. For instance, terpenes are the principal volatile substances in propolis (constituting 10% of total) and contribute to its antioxidant and antimicrobial actions (Huang et al. 2014). Phenolics also contribute to the antibacterial and anti–trypanosomal activities of propolis (Marcucci et al. 2001).

**TABLE 2:** Common flavonoids identified in propolis and their biological activities.

Flavonoid	Geographical source	Biological activities	References
Apigenin	Italy	Antioxidant	(Gregoris and Stevanato 2010)
Chrysin	Italy	Antioxidant	(Gregoris and Stevanato 2010)
Formononetin	Brazil	Antifungal	(Freires et al. 2016; das Neves et al. 2016)
Hesperetin	Mexico	Anti‐ *Giardia lamblia*	(Alday‐Provencio et al. 2015)
Kaempferol	Italy	Antioxidant	(Gregoris and Stevanato 2010)
	Brazil	Antifungal	(Freires et al. 2016)
Medicarpin	Brazil	Antifungal	(Freires et al. 2016)
Naringenin	Italy	Antioxidant	(Gregoris and Stevanato 2010)
	Mexico	Anti‐ *Giardia lamblia*	(Alday‐Provencio et al. 2015)
Neovestiol	Brazil	Antibacterial Anti‐inflammatory	(Bueno‐Silva et al. 2013)
Pinocembrin	Italy	Antioxidant	(Gregoris and Stevanato 2010)
	Mexico	Anti‐ *Giardia lamblia*	(Alday‐Provencio et al. 2015)
Quercetin	Italy	Antioxidant	(Gregoris and Stevanato 2010)
	Brazil	Antifungal	(Freires et al. 2016)
Vestitol	Brazil	Antibacterial Anti‐inflammatory	(Bueno‐Silva et al. 2013)
		Antifungal	(Freires et al. 2016)
Galangin	China	Antioxidant	(Nie et al. 2013; Caruso et al. 2022)

*Note:* This table is adapted from Santos et al. (2020).

The use of propolis dates back to 300 bc, when it was employed by the Egyptians, Greeks, and Romans as a medicinal substance for the treatment of skin lesions and other conditions (Sforcin 2007). It is well recognized that propolis and its constituents have diverse biological activities and clinical applications such as antioxidant, anti‐tumor, antimicrobial, and anti‐inflammatory properties (Forma and Bryś 2021; Zullkiflee et al. 2022; Almuhayawi 2020). In recent years, propolis has been developed as a complementary medicine because of these potentially valuable clinical actions (Castaldo and Capasso 2002). For example, clinical studies have demonstrated the potential antioxidant and anti‐inflammatory effects of propolis in breast cancer patients undergoing chemotherapy (Darvishi et al. 2020). Additionally, clinical research has found that propolis can attenuate inflammatory markers in patients undergoing hemodialysis (Chermut et al. 2023). Attempts to evaluate its chemical composition and medicinal properties have been made (Król et al. 2013). For example, the caffeic acid phenethyl ester (CAPE) found in propolis has been demonstrated to have antioxidant (Russo et al. 2002), anticancer (Kabała‐Dzik et al. 2017), and anti‐inflammatory activities (Khan et al. 2007), as well as potential therapeutic actions in neurological disorders (Menezes da Silveira et al. 2021). Moreover, the diterpenoid compounds present in propolis have been shown to possess antimicrobial (El‐Guendouz et al. 2016), anticancer (Tazawa et al. 2016), and antiparasitic activity (Siheri et al. 2014).

Batch‐to‐batch variation is a common issue encountered in the manufacture of propolis extracts. The active components of the standardized Brazilian green propolis extract EPP‐AF include the flavonoids aromadendrin and isosakuranetin, and the phenolic agents caffeic acid, p‐coumaric acid, cinnamic acid, and artepillin C (Berretta et al. 2012; de Castro et al. 2011). However, although EPP‐AF has anti‐inflammatory (Hori et al. 2013; Duarte Silveira et al. 2022), antibacterial (Berretta et al. 2013; Rebouças‐Silva et al. 2023), and antioxidant (Diniz et al. 2020) actions, the potential roles of constituent flavonoids are unclear. Studies using individual flavonoids could clarify these pharmacological actions.

Flavonoids are important constituents of many natural products (Feliciano et al. 2015; Kurek‐Górecka et al. 2014) and elicit a range of pharmacological actions, including anti‐oxidative, anti‐inflammatory, and anti‐cancer benefits (Romano et al. 2013). Although flavonoids are not unique to propolis, there are reports that bee products, including propolis, have anti‐angiogenesis actions that are beneficial in the treatment of human diseases (Izuta et al. 2009; Li et al. 2021; Shaker et al. 2023; Mehany et al. 2022). A number of flavonoids found in propolis have been shown to possess clinical activity. For instance, the propolis flavonoid chrysin has been shown to modulate nuclear factor‐kappa B (NF‐κB) expression and the production of reactive oxygen species (ROS) in cells in vitro, which contributes to anti‐inflammatory and antioxidant actions (Mantawy et al. 2014). The antioxidant properties of galangin can mitigate oxidative stress in diabetic rats, suggesting its therapeutic potential in the management of diabetes (Aloud et al. 2017). It has also been demonstrated that flavonoids extracted from propolis are potent in the treatment of angiogenesis‐driven diseases (Ahn et al. 2009; Zhao et al. 2016). Such effects may be mediated by the inhibition of VEGF signaling via the HIF‐1α (Fu et al. 2007), STAT3 (Ansó et al. 2010) and Akt/PKB pathways (Huang et al. 2015) in intraocular neovascular syndromes (Gupta et al. 2013; Smith and Kaiser 2014; Zhuang et al. 2011), solid tumours (Madu et al. 2020; Hwang and Heath 2010; Muto et al. 2015), inflammation (Jackson et al. 1997) and other conditions. Importantly, propolis flavonoids are also able to influence angiogenesis via VEGF‐independent pathways. For instance, luteolin, a flavone found in propolis, suppresses Notch‐1 signaling in MDA‐MB‐231 cells and decreases breast cancer cell proliferation and angiogenesis in xenografted mice (Sun et al. 2015).

## 4 Flavonoids Isolated From Propolis Influence Angiogenesis in Intraocular Neovascular Syndromes

Intraocular neovascularization can be divided into retinal neovascularization that can lead to diabetic retinopathy (DR) and choroidal neovascularization that may result in AMD (Zhou et al. 2017). As mentioned earlier, although anti‐VEGF drugs can inhibit intraocular neovascularization, the adverse reactions they often trigger remain a significant concern. To identify safer and more effective anti‐angiogenic agents for retinal diseases, preclinical studies have investigated flavonoids that are present in propolis for their efficacy in common retinal diseases associated with intraocular neovascularization (Table 3).

**TABLE 3:** The major flavonoids in propolis that have potential anti‐angiogenic actions against retinal diseases associated with intraocular neovascularization.

Retinal disease	Flavonoid	Experimental model	Concentration used	References
Diabetic retinopathy	Chrysin	Primary human retinal microvascular endothelial cells	1–20 μM	(Kang et al. 2016)
		db/db C57BLKS/+ Lepr^db^ Iar mice	10 mg/kg	
		Rhesus macaque choroid‐retinal (chorioretinal) endothelial RF/6A cells	3 μM, 10 μM, 30 μM	(Liao et al. 2020)
	Kaempferol	Human retinal endothelial cells	5 μM, 25 μM	(Xu et al. 2017)
	Quercetin	Streptozotocin‐induced diabetic Sprague–Dawley rats	150 mg/kg	(Chen et al. 2017)
	Hesperetin	Streptozotocin‐induced diabetic Wistar albino rats	200 mg/kg	(Kumar et al. 2012)
Wet type of Age‐related macular degeneration	Quercetin	Rhesus macaque choroid‐retinal (chorioretinal) endothelial RF/6A cells	10 μM, 50 μM, 100 μM	(Li et al. 2015)
	Chrysin	Laser‐induced CNV brown norway rats	60 mM	(Song et al. 2020)
		Human ARPE‐19 cells	3 μM	(Wang et al. 2024)
		Laser‐induced CNV C57BL/6J mice	25 mg/kg	
	Formononetin	Human ARPE‐19 cells	0.2 μM, 1 μM, 5 μM	(Wu et al. 2016)

### 4.1 Diabetic Retinopathy

DR is a major complication of diabetes (Yau et al. 2012; Thomas et al. 2019). The worldwide incidence of DR was over 103 million in 2020 and could exceed 160 million by 2045 (Teo et al. 2021). Although the pathogenesis of DR remains unclear, it has been acknowledged that oxidative stress, inflammatory responses, and enhanced angiogenesis due to hyperglycemia or glucose intolerance are primary risk factors (Cheng et al. 2019; Kowluru and Chan 2007; Obrosova and Kador 2011; Abu El‐Asrar et al. 2009).

DR can be categorized into two clinical stages: non‐proliferative and proliferative (Wang and Lo 2018), that are distinguished by the presence or absence of abnormal neovascularization (Chaudhary et al. 2021). Patients with proliferative DR may experience vision impairment, characterized by vitreous hemorrhage due to bleeding from new abnormal vessels or the development of tractional retinal detachment (Wang and Lo 2018). Elevated VEGF is widely observed in the vitreous humor and fibrovascular tissues of patients with proliferative DR (Aiello et al. 1994; Chung et al. 2011; Pe'er et al. 1996). Thus, VEGF inhibition to decrease neovascularization and vessel leakage is a potential treatment for DR (Gupta et al. 2013).

In retinal angiogenesis, retinal vascular endothelial cells proliferate, penetrate the internal limiting membrane, and extend into the vitreous humor, leading to complications such as vitreous hemorrhage and tractional retinal detachment (Kvanta 2006). Chrysin has been reported to relieve the overexpression of HIF‐1α and VEGF induced by high glucose concentrations in human retinal microvascular endothelial cells and in mice in vivo (Kang et al. 2016). This suggests that chrysin may alleviate retinal neovascularization caused by retinal hypoxia due to chronic hyperglycemia (Kang et al. 2016). These findings are in accord with the effects of high glucose concentrations on chorioretinal endothelial cells (Liao et al. 2020). Kaempferol was also anti‐angiogenic in human retinal endothelial cells and inhibited the activation of migration and tube formation by high glucose. The mechanism underpinning these actions of kaempferol involves the suppression of VEGF and placental growth factor (Xu et al. 2017). The suppression of VEGF was also demonstrated in diabetic rats that were treated with quercetin (Chen et al. 2017). Similarly, in streptozotocin‐induced diabetic rats, the oral administration of hesperetin attenuated vessel dilation and vascular dysfunction compared to control (Kumar et al. 2012). Furthermore, retinas of rats treated with hesperetin had reduced VEGF expression compared to the untreated group (Kumar et al. 2012). Together, these findings suggest that flavonoids such as kaempferol, chrysin, quercetin, or hesperetin could be developed as angiogenesis‐targeting agents for DR.

### 4.2 Wet Type Age‐Related Macular Degeneration

In developed countries, AMD is the leading cause of irreversible central vision loss (Ferris et al. 2013; Steinmetz et al. 2021). AMD is closely associated with complex interactions of metabolic, functional, genetic, and environmental factors within the ocular structures of the macular region, such as the choroid, Bruch's membrane, retinal pigment epithelium, and photoreceptors (Nowak 2006). AMD is characterized by pathological changes affecting the deeper retinal layers of the macula and the surrounding vasculature, ultimately leading to the loss of central vision (Thomas et al. 2021). There are two types of AMD: wet and dry AMD (Serener and Serte 2019). Dry AMD, or atrophic AMD, is characterized by dysfunction of the retinal pigment epithelium, loss of photoreceptors, and degeneration of the retina (Schultz et al. 2021). Wet AMD, or neurovascular AMD, is characterized by anomalous choroidal neovascularization (CNV) within the central region of the retina (Smith and Kaiser 2014; Zhuang et al. 2011). Endothelial permeability is the first step of angiogenesis in which endothelial cell–cell junctions are loosened so that new sprouts may develop from these vessels. Because the newly formed vessels lack an outer layer, they are often leaky. Mechanistically, phosphorylation of VEGFR2 sequentially stimulates c‐SRC and VE‐cadherin proteins that promote angiogenesis and vascular leakage. The formation of neovascular tissues and the accumulation of exudates result in macular scars and lead ultimately to central blindness (Al‐Khersan et al. 2019). VEGF‐A has been recognized as the key regulator in the advancement of CNV in wet AMD (Reid et al. 2018). The front‐line treatment for wet AMD involves recurrent intravitreal administration of VEGF‐A antagonists (Reid et al. 2018).

The therapeutic value of flavonoids in the treatment of wet AMD is primarily attributed to the inhibition of angiogenesis. In a rat model, the intravitreal injection of chrysin decreased the thickness of CNV lesions in neovascular tissues (Song et al. 2020). Additionally, the expression of VEGF and HIF‐1α was markedly decreased by chrysin (Song et al. 2020). Similar observations were made by Jing et al. in ARPE‐19 cells and in a laser‐induced CNV mouse model (Wang et al. 2024), where CNV lesions were decreased by chrysin, while STAT3 and VEGF‐A expression were decreased, suggesting that chrysin may inhibit angiogenesis by modulating VEGF signaling via STAT3 deactivation (Wang et al. 2024). Quercetin also inhibited VEGF‐induced migration and tube formation in choroid‐retinal endothelial cells from rhesus macaque; signaling downstream from VEGFR‐2 was also attenuated (Li et al. 2015).

In hypoxic ARPE‐19 cells, the flavonoid formononetin effectively suppressed the expression of VEGF and HIF‐1α proteins (Wu et al. 2016). Additionally, formononetin inhibited retinal neovascularization, vessel tortuosity, and vessel dilation in a rat model of oxygen‐induced retinopathy by targeting the HIF‐1α/VEGF signaling pathway (Wu et al. 2016).

Taken together, essential flavonoids present in propolis show promise for the treatment of DR and wet AMD by inhibiting intraocular neovascularization. However, therapeutic safety information (e.g., toxicity profiles and adverse effects) for such flavonoids is now required because other anti‐VEGF agents have been found to produce adverse effects.

## 5 Flavonoids Isolated From Propolis Modulate Angiogenesis in Cancers

Angiogenesis is a key cellular event in cancer progression and metastasis. The progression of solid tumours is regulated by the microenvironment (Chen et al. 2019). As mentioned, angiogenesis is important for the supply of oxygen, nutrients, and immune cells to the tumour microenvironment, and also for waste removal. Neovascularization also provides a pathway for the dissemination of malignant cells by metastasis (Makrilia et al. 2009). Therefore, the inhibition of angiogenesis is a promising strategy for the treatment of cancer.

Preclinical studies suggest that flavonoids from propolis may have value in the treatment of solid tumours (Table 4). Thus, flavonoids that are enriched in propolis exhibit potent anti‐angiogenic activities in breast cancer, prostate cancer, and hepatocellular carcinoma (HCC). However, most research that has investigated the anti‐angiogenic effects of flavonoids in solid tumours is based on preclinical studies. The available information on clinical outcomes with flavonoids in cancer treatment and whether they produce any adverse effects is sparse.

**TABLE 4:** Major flavonoids that are present in propolis and that have potential anti‐angiogenic effects against solid tumors.

Solid tumour	Flavonoid	Experimental model	Concentration used	References
Breast cancer	Quercetin	DU145 and HEK293T cells	10 μM, 30 μM, 50 μM	(Zhao et al. 2016)
		7,12‐dimethylbenzanthracene (DMBA)‐induced mammary carcinoma Sprague–Dawley rats	3% of the diet weight	(Kong et al. 2005)
		MCF‐7 and MDA‐MB‐231 cells	50 μM, 100 μM	(Balakrishnan et al. 2016)
	Galangin	MCF‐7 and MDA‐MB‐231 cells	50 μM, 100 μM	(Qaddoori and Al‐Shmgani 2023)
Prostate cancer	Apigenin	PC3‐M cells	25 μM, 50 μM, 100 μM	(Mirzoeva et al. 2008)
	Chrysin	PC3‐M cells	10 μM	(Han et al. 2022)
	Quercetin	PC3‐M cells and HUVECs	40 μM	(Pratheeshkumar et al. 2012; Ma et al. 2004)
		Human prostate tumour xenograft mice	20 mg/kg	
		CWR22 prostate tumour xenograft mice	200 mg/kg	(Ma et al. 2004)
Hepatocellular carcinoma	Quercetin	Diethylnitrosamine and 2‐acetylaminofluoren‐induced hepatocellular carcinoma rat model	50 mg/kg	(Abdu et al. 2022)
		HepG2 cells	107.7 μM	
	Kaempferol	Huh7 cells	1 μM, 5 μM, 10 μM, 50 μM	(Mylonis et al. 2010)
	Apigenin	NK‐92 and SK‐Hep1 cells	50 μM	(Lee and Cho 2022)
		Huh7 cells	40 μM, 80 μM, 120 μM	(Kim et al. 2011)

### 5.1 Breast Cancer

Breast cancer is the most common and lethal cancer in women (Bray et al. 2018; Murray and Lopez 1997; Sharma et al. 2010; Akram et al. 2017). The 5‐year relative survival rates of breast cancer differ significantly between developed (~60%) and developing countries (~40% or less) (Coleman et al. 2008), with resource constraints being a primary underlying reason (Anderson et al. 2008). Metastatic disease is a lethal feature of breast cancer and begins with local invasion of primary tumor cells into surrounding tissues. This is followed by intravasation of tumor cells into blood or lymphatic vessels and transport to distant organs (Scully et al. 2012).

Angiogenesis is a key determinant of the supply of oxygen and nutrients that supports the expansion of breast cancer (Madu et al. 2020). Because the VEGF/VEGFR axis has an important role in breast tumor angiogenesis (Linardou et al. 2012), the inhibition of this pathway could be a promising approach in the treatment of the disease (Longatto Filho et al. 2010). In a rat model of breast cancer, quercetin significantly decreased tumor growth by ~30% and tumor micro‐vessel density by ~40%, which is consistent with a moderate inhibitory effect on tumor neovascularization and progression (Kong et al. 2005). Decreased expression of VEGF was noted, which may contribute to the anti‐cancer and anti‐angiogenic effect of quercetin (Kong et al. 2005). Moreover, in female nude mice that carried ER‐positive breast cancer xenografts, Zhao et al. (2016). Found that intragastric quercetin administration decreased the expression of VEGF and VEGFR2 in tumours (Zhao et al. 2016).

The use of innovative delivery systems could enhance the anti‐angiogenic efficacy of flavonoids. Thus, gold nanoparticle–conjugated quercetin decreased the migration and invasion activities of ER‐positive and ER‐negative breast cancer cells, possibly due to decreased expression of VEGFR2 and the attenuation of downstream signaling (Balakrishnan et al. 2016). Compared to free quercetin, gold nanoparticle–conjugated quercetin decreased VEGFR2 expression and neovascularization in human umbilical vein endothelial cells more effectively (Balakrishnan et al. 2016). Gold nanoparticle–conjugated galangin also inhibited angiogenesis in breast tumours (Qaddoori and Al‐Shmgani 2023). It was noted that free galangin significantly decreased the migration and the extent of angiogenesis in MCF‐7 and MDA‐MB‐231 cells, while gold nanoparticles exert their function by inhibiting the phosphorylation of ERK1, VEGF, and VEGFRs. The downregulation of ERK1 and VEGF inhibited the expression of genes involved in angiogenesis, migration, and cell cycle progression (Qaddoori and Al‐Shmgani 2023).

Clinical studies with these flavonoids have produced unsatisfactory outcomes. Although quercetin showed anti‐cancer activity in a phase I clinical trial (Ferry et al. 1996), a study of 353 breast cancer patients and 701 healthy controls found no association between the consumption of tea polyphenols and flavanols (e.g., quercetin and kaempferol) and decreased breast cancer risk (Luo et al. 2010). However, there is some evidence that propolis may have clinical benefits in breast cancer management as an adjuvant therapy to decrease the adverse effects of chemotherapy or radiotherapy and in improving the quality of life and nutritional status (Darvishi et al. 2020; Ebeid et al. 2016; Piredda et al. 2017; Davoodi et al. 2022).

### 5.2 Prostate Cancer

Prostate cancer is a significant public health concern, with over 1.1 million cases identified globally every year (Lam et al. 2019). Although androgen deprivation therapy is the major treatment, there are concerns due to adverse effects and disease relapse (Cannata et al. 2012; Yap et al. 2016). Prostatic intraepithelial neoplasia (PIN) is characterized by the abnormal growth of epithelial cells within normal prostatic acini or ducts (Kim and Yang 2002). High‐grade PIN is widely acknowledged as a precursor to prostate cancer (Brawer 2005). Similar to other tumors, PIN co‐opts nearby blood vessels, which leads to the regression of these vessels and promotes angiogenesis at the tumour edge (Bostwick and Qian 2004). Suppression of angiogenesis is a promising strategy for prostate cancer, especially in individuals with elevated risk of high‐grade PIN (Bostwick and Qian 2004).

Several of the flavonoids that are present in propolis may be effective against prostate cancer. Apigenin decreased hypoxia‐induced activation of HIF‐1α and Akt pathways in metastatic prostate cancer cells (Mirzoeva et al. 2008), by decreasing both the stability of HIF‐1α protein and down‐regulating steady state HIF‐1α mRNA in normoxic and hypoxic conditions (Mirzoeva et al. 2008). Because VEGF transcription largely relies on HIF‐1α in hypoxia, apigenin decreases VEGF expression which, in turn, decreases angiogenesis in prostate cancer (Mirzoeva et al. 2008). Chrysin also decreases VEGF and HIF‐1α expression in metastatic prostate cancer cells in hypoxia (Han et al. 2022); however, its clinical application may be limited by poor systemic bioavailability (Gao et al. 2021). Quercetin can also inhibit VEGF secretion by prostate cancer cells and attenuate AKT/mTOR/P70S6K signaling (Pratheeshkumar et al. 2012). It has also been demonstrated that quercetin can inhibit angiogenesis by decreasing VEGFR2 expression on the surface of endothelial cells (Pratheeshkumar et al. 2012). In severe combined immunodeficiency mice bearing CWR22 prostate tumor cell xenografts, the combination of tamoxifen and quercetin had a more pronounced inhibitory effect on VEGF expression than tamoxifen or quercetin alone (Ma et al. 2004).

There have been very few clinical trials of flavonoids in prostate cancer management. A randomized controlled double‐blind crossover trial was initiated in 2012 (clinical trial ID: NCT01538316) that aimed to investigate the prophylactic effectiveness of quercetin and genistein in individuals at higher prostate cancer risk. However, no results have yet been reported.

### 5.3 Hepatocellular Carcinoma

HCC ranks sixth in global cancer incidence and is the third leading cause of cancer‐related mortality due to its dismal prognosis (Parkin et al. 2005). The primary risk factors associated with HCC include hepatitis B or C virus infection, alcoholic cirrhosis, and non‐alcoholic fatty liver disease (Bruix et al. 2014). There is an urgent need to develop novel and effective systemic therapies for HCC, largely due to the unfavorable prognosis in patients with advanced disease and the high recurrence rate after surgical resection (Zhu 2010). In the clinical setting, hypervascularity is a prominent characteristic of HCC (Muto et al. 2015). Moreover, VEGF is a crucial angiogenic factor for the development of HCC. Several anti‐angiogenic agents have been approved for clinical use in HCC, including the anti‐VEGF antibody bevacizumab that is co‐administered with the anti‐PD‐L1 antibody atezolizumab. Other anti‐angiogenic agents are undergoing clinical trials, including the multi‐kinase inhibitor regorafenib that targets VEGFR1–3 and several other receptors (Ruff and Pawlik 2024). Therefore, disrupting VEGF signaling via drug treatment or in conjunction with locoregional therapy may be promising therapeutic options for HCC (Finn and Zhu 2009).

Quercetin decreased the expression of VEGF in a rat model of chemically‐induced HCC, which was accompanied by decreased angiogenesis and proliferation (Abdu et al. 2022). Furthermore, the combination of quercetin and sorafenib, which was the first drug approved for the treatment of HCC, was more active than the individual drugs (Abdu et al. 2022). The flavonoid kaempferol deactivates HIF‐1α signaling by promoting its relocation from the nucleus to the cytoplasm in hypoxic human hepatoma cells, thereby inhibiting VEGF transcription, which suppressed angiogenesis and malignant transformation (Mylonis et al. 2010). Similarly, apigenin increased the cytotoxicity of natural killer cells against HCC by reducing HIF‐1α expression (Lee and Cho 2022). Moreover, apigenin reduced VEGF and MMP‐8 expression, which suppressed angiogenesis and migration and promoted apoptosis in human hepatoma cells (Kim et al. 2011). Jin et al. showed that CAPE is a potent inhibitor of MMP‐9 function in human Hep3B cells and primary mouse hepatocytes. Thus, CAPE has a potential role in the suppression of tumor invasion and metastasis (Jin et al. 2005).

## 6 Flavonoids Isolated From Propolis Regulate Angiogenesis in Other Diseases

Angiogenesis is also associated with other pathological processes, such as inflammation and cardiovascular diseases (CVDs). As mentioned, angiogenesis triggers the formation of new blood vessels by activating the release of angiogenic factors. Alterations in vessel walls favor the migration and proliferation of endothelial cells and promote tube formation. The rate of angiogenesis is regulated by the interplay of pro‐angiogenic, angiostatic, and anti‐angiogenic factors, including VEGF. Thus, flavonoids that have anti‐angiogenic activity may be useful in the treatment of angiogenesis‐driven inflammatory diseases and CVDs.

### 6.1 Inflammation

Inflammation is a critical response to biological, chemical, and physical stimuli (Germolec et al. 2018). Acute inflammation is an important mechanism in tissue repair following injury, but prolonged inflammation may contribute to the development of chronic disease (Pahwa et al. 2018). Inflammation is regulated by multiple cells, mediators, and signaling mechanisms. The pro‐inflammatory cytokines interleukin 1β (IL‐1β), TNF‐α, and interleukin‐6 (IL‐6) play key roles in inflammation (Hirano 2021). For example, IL‐6 regulates lymphocyte differentiation, cell proliferation and survival, and the suppression of apoptotic signals (Hodge et al. 2005). IL‐6 is also a pivotal mediator in numerous autoimmune and chronic inflammatory disorders such as rheumatoid arthritis (RA), diabetes, and Crohn's disease (Neurath and Finotto 2011). TNF‐α and IL‐6 have been shown to induce angiogenesis by upregulating VEGF expression (Nagineni et al. 2012; Forooghian and Das 2007; Al‐Rasheed et al. 2013; Wang et al. 2016). Newly formed blood vessels transport inflammatory cells to the site of inflammation, thereby sustaining the chronic inflammatory state, and provide nutrients and oxygen for tissue proliferation (Jackson et al. 1997). Therefore, attenuation of TNF‐α and IL‐6‐mediated VEGF activation is a potential approach to decrease chronic inflammation and suppress angiogenesis. Flavonoids that have anti‐angiogenic properties, such as chrysin, quercetin, galangin, naringenin, and medicarpin, have been investigated for their anti‐inflammatory effects.

The process of cell migration and tube formation in lipopolysaccharide (LPS)‐induced angiogenesis in human umbilical vein endothelial cells (HUVECs) can be inhibited by chrysin (Lin et al. 2006). Chrysin decreased IL‐6 and IL‐6R expression in HUVECs and dysregulated the VEGF/VEGFR‐2 pathway, which suppressed LPS‐induced angiogenesis (Lin et al. 2006). In a rat model of renal injury, quercetin decreased the expression of pro‐inflammatory serum markers, such as TNF‐α, IL‐6, and C‐reactive protein, as well as VEGF (Al‐Rasheed et al. 2013). Similarly, in LPS‐treated fibroblast‐like synovial cells, galangin decreased the production of inflammatory markers like TNF‐α, IL‐1β, and IL‐6 in a concentration‐dependent manner (Deng et al. 2022). In accord with these findings, PI3K/AKT signaling downstream from VEGF was markedly decreased by galangin in a rat model of RA (Deng et al. 2022). Thus, galangin may disrupt the progression of RA by inhibiting the Akt/PKB pathway and attenuating the production of inflammatory molecules (Deng et al. 2022). Treatment with naringenin decreased VEGF production and inhibited capillary formation by HUVECs (Li et al. 2016). Moreover, the expression of IL‐6 in HUVECs was suppressed by naringenin. Thus, naringenin could have a potential role in modulating the inflammatory response in order to elicit anti‐angiogenic and anti‐cancer effects (Li et al. 2016). In a mouse model of postmenopausal arthritis, medicarpin suppressed VEGF and IL‐1β expression and prevented cartilage erosion and bone resorption (Mansoori et al. 2020). Additionally, the serum concentrations of the pro‐inflammatory cytokines TNF‐α and IL‐6 were suppressed by medicarpin, thus demonstrating its potential in preventing cartilage degradation and bone resorption (Mansoori et al. 2020).

An open label Phase 1 pilot study conducted in patients with diabetic kidney disease found that quercetin and dasatinib prevented the release of pro‐inflammatory factors by decreasing the senescent cell burden in adipose tissue (Hickson et al. 2019). Another study found that quercetin modulated iron status in patients with thalassemia, although its capacity to modulate iron‐associated inflammation remained uncertain (Sajadi Hezaveh et al. 2019). A meta‐analysis of multiple clinical studies suggested that quercetin supplementation decreased circulating levels of the inflammatory biomarker, C‐reactive protein, provided the dose exceeded 500 mg/day and C‐reactive protein was lower than 3 mg/L (Mohammadi‐Sartang et al. 2017). In a randomized double‐blind clinical trial in patients with rheumatoid arthritis, quercetin decreased serum TNFα and inflammatory symptoms (Javadi et al. 2017).

### 6.2 Cardiovascular Diseases

CVDs include hypertension, ischemic stroke, atherosclerosis, coronary heart disease, cerebrovascular disease, and rheumatic heart disease (Modi et al. 2012). An unresolved problem in CVDs is the formation of new blood vessels, which contributes to disease pathogenesis. However, as mentioned, dysregulated angiogenesis in pathological conditions produces new blood vessels that are structurally abnormal. Thus, therapeutic regulation of angiogenesis could be a viable approach to promote normal blood vessel development in CVDs; this could decrease the risks associated with percutaneous coronary intervention or artery bypass surgery (Deveza et al. 2012). In addition, oxidative stress and obesity are important risk factors for CVDs. The promising antioxidant and anti‐obesity properties of flavonoids and other polyphenols could contribute to the cardioprotective actions of propolis (Chavda et al. 2024).

Endothelial progenitor cells are frequently impaired in patients who are at risk of atherosclerosis. The 5,7‐dihydroxyflavone derivative Pinocembrin is present in propolis. Pinocembrin promotes the differentiation, proliferation, and tube formation of endothelial progenitor cells in vitro in cultured rat bone marrow‐derived EPCs and in vivo in the apoE^−/−^ mouse model. The mechanism likely involves the modulation of the PI3K/eNOS/NO signaling cascade (Yang et al. 2013).

Zhao et al. evaluated the neuroprotective effects of the flavonoid rutin and hyaluronic acid in a co‐culture system containing rat brain microvascular endothelial cells and microglial cells, a rat model of acute ischemic stroke‐reperfusion injury, a mouse model of cerebral vessel occlusion, and zebrafish, using an advanced nano delivery system (Zhao et al. 2023). Neurological damage after stroke was relieved by improving angiogenesis in the penumbra and enhancing neovascularization. Sodium formononetin‐3′‐sulphonate, a derivative of formononetin, showed neuroprotective activity in a rat model of ischemia and reperfusion injury and improved cerebrovascular angiogenesis in HUVECs by increasing the expression of VEGF and platelet endothelial cell adhesion molecule 1 (an endothelial cell marker) (Zhu et al. 2014).

In summary, preclinical studies have demonstrated potential anti‐inflammatory and cardioprotective benefits of flavonoids present in propolis. However, there is insufficient evidence to support its clinical value at this stage. Future research could explore the combination of flavonoids with common anti‐inflammatory and cardiovascular drugs to enhance the anti‐angiogenic effects. Alternately, the use of novel drug delivery carriers could enhance the delivery of flavonoids to tissues and improve their clinical utility.

## 7 Conclusions

VEGF‐induced angiogenesis is a leading risk factor for several cancers (Rosen 2002), intraocular neovascular syndromes (Marneros 2016) and inflammation (Jackson et al. 1997). Flavonoids in propolis, such as chrysin and quercetin, have the potential to inhibit this process (Lin et al. 2006, 2010; Wang et al. 2020). However, clinical data are now required to support the application of propolis flavonoids in the treatment of angiogenesis‐driven diseases. It should be added that there are other flavonoids present in propolis, such as vestitol and neovestiol, that have not yet been extensively studied for their anti‐angiogenic effects. These and other chemicals are potential contributors to the beneficial effects of propolis and its products, the synergistic effects of which are yet to be evaluated.

An improved understanding of the efficacy, safety, and pharmacokinetics of propolis flavonoids is also essential for future clinical translation. Indeed, an adverse feature of some natural compounds is their limited water solubility that can lead to suboptimal bioavailability (Sauter 2020). Structure–activity relationships and detailed mechanisms by which flavonoids in propolis modulate angiogenesis remain largely unclear. To date, experimental and in silico analysis suggest that hydroxylation of the A ring, especially at positions 5 and 7, and the B ring, especially at the 4′ position, could be important for the regulatory actions of flavonoids on VEGF (Ansó et al. 2010; Gacche et al. 2011). In terms of their mode of action, some flavonoids, like apigenin, decrease VEGF by suppressing the transcription factor HIF‐α but others, such as luteolin and quercetin, exert their inhibitory effect on VEGF by inducing HIF‐α (Ansó et al. 2010; Luo et al. 2008). This suggests that HIF‐α is not the major regulatory factor. Interestingly, the VEGF inhibitory potencies of the flavonoids that have been evaluated appear to correlate with inhibition of the angiogenic transcription activator, Signal transducer and activator of transcription 3 (STAT3). However, these studies are based on a limited number of flavonoids, and future research should include a larger number of chemically diverse flavonoids to expand the findings.

Advanced drug delivery approaches such as electrochemotherapy, liposomes, nanoparticles, and nano‐micelles may be employed to promote the clinical applications of flavonoids present in propolis (Afra et al. 2020; Dutta et al. 2019; Cheng et al. 2022). For example, solid lipid nanoparticles have been shown to improve delivery and stabilize active substances against metabolic and physical degradation (Müller et al. 2000). It has also been suggested that propolis‐loaded solid lipid nanoparticles demonstrate improved efficacy (Taherzadeh et al. 2021). This could be a new direction in the application of propolis flavonoids in treatment. However, it is important to note that the regulatory approval and commercial availability of flavonoids vary between countries and that some flavonoids, such as apigenin, are approved as dietary supplements (Sharma et al. 2022). A detailed discussion of the regulatory status of flavonoids in different countries is beyond the scope of this review.

Overall, certain flavonoids that are enriched in propolis have shown promising anti‐angiogenic actions due to their capacity to modulate VEGF signaling and could be developed as novel therapeutics to target multiple angiogenesis‐driven diseases. It is now important that future research explores the clinical potential of propolis flavonoids in disease management.

## Author Contributions

**Yipeng Lin:** data curation, formal analysis, methodology, writing – original draft. **Janney Z. Wang:** formal analysis, writing – review and editing. **Yihe Niu:** formal analysis, writing – review and editing. **Ling Zhu:** formal analysis, writing – review and editing. **Rujee K. Duke:** formal analysis, writing – review and editing. **Colin C. Duke:** formal analysis, writing – review and editing. **Michael Murray:** formal analysis, writing – review and editing. **Fanfan Zhou:** conceptualization, data curation, formal analysis, funding acquisition, investigation, methodology, project administration, resources, software, supervision, validation, visualization, writing – review and editing.

## Conflicts of Interest

The authors declare no conflicts of interest.

## Data Availability

All literature mentioned in this review are available through the journal's websites.
